# Cardiomyopathie du péripartum: à propos d’une observation et revue de la littérature

**DOI:** 10.11604/pamj.2016.25.21.10372

**Published:** 2016-09-26

**Authors:** Abdelmajid Bouzerda

**Affiliations:** 1 Premier Centre médicochirurgical, Faculté de Médecine et de Pharmacie de Marrakech, Maroc

**Keywords:** Cardiomyopathie du péripartum, insuffisance cardiaque, grossesse, Peripartum cardiomyopathy, heart failure, pregnancy

## Abstract

La cardiomyopathie du péripartum ou cardiomyopathie gravidique primitive, est une entité rare et méconnue définie comme une insuffisance cardiaque systolique survenant le dernier mois de la grossesse ou les cinq premiers mois du postpartum en l'absence d'étiologie connue ou de cardiopathie préexistante. Nous rapportons l'observation d'une jeune patiente de 31 ans sans antécédents pathologiques particuliers admise pour une poussée d'insuffisance cardiaque gauche 1mois après son accouchement. Le contexte de péripartum nous a fait penser au diagnostic et a motivé la réalisation d'une Echocardiographie qui a confirmé cette entité.

## Abstract (en)

Peripartum cardiomyopathy or primitive pregnancy-induced cardiomyopathy is a rare and little known entity characterized systolic heart failure occurring in the last month of pregnancy or in the first five months postpartum in the absence of known cause or pre-existing heart disease. We report the case of a 31-year old young patient with no significant past medical history admitted to hospital with congestive left heart failure 1 month after delivery. Presumptive diagnosis was made on the basis of the context of peripartum and justified the realization of CT scan which confirmed this entity.

## Introduction

La cardiomyopathie du péripartum est une cause rare de cardiomyopathie dilatée survenant en fin de grossesse ou dans les mois suivant l'accouchement. Le diagnostic repose sur l'association d'un tableau d'insuffisance cardiaque clinique et d'une dysfonction systolique ventriculaire gauche en échocardiographie. Bien que plusieurs hypothèses physiopathologiques aient été avancées, les causes exactes de cette affection restent inconnues. L'évolution est imprévisible, parfois favorable avec une rémission complète, mais souvent il y a une persistance ou une aggravation de l'insuffisance cardiaque pouvant être délétère. Le risque de récurrence lors d'une grossesse ultérieure malgré une rémission apparente, est très élevé.

## Patient et observation

**Figure 1:**
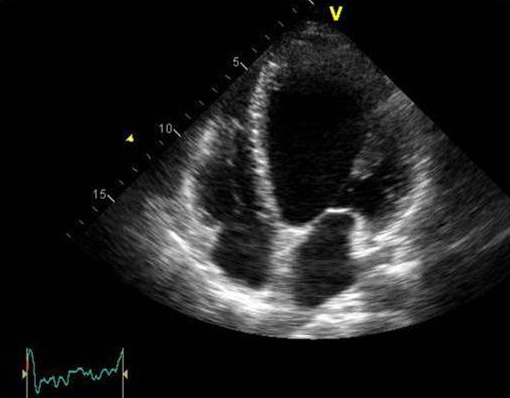
Incidence apicale des 4 cavités montrant un VG dilaté et globuleux

**Figure 2:**
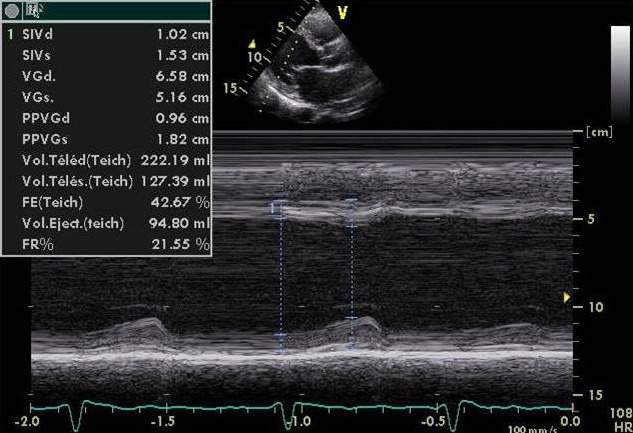
Coupe TM sur le VG en incidence parasternale grand axe montrant une dilatation et une dysfonction systolique ventriculaire gauche

Mme D.L, âgée de 31 ans, est hospitalisée en unité de soins intensifs cardiologiques suite à une poussée d'insuffisance cardiaque gauche 01 mois après un accouchement normal par voie basse. La patiente est à 38 semaines d'aménorrhée lors de l'accouchement, et la grossesse s'est déroulée normalement. Elle n'a pas d'antécédents médicaux particuliers, notamment pas d'antécédents cardiaque. A l'admission la patiente est orthopneique Tachycarde à 120 bpm avec une TA à 100/ 65 mmhg. L'auscultation cardiaque retrouve un Souffle systolique d'insuffisance mitrale coté 2/6 associé à un galop gauche protodiastolique L'auscultation pleuropulmonaire note des râles crépitants aux hemichamps pulmonaires Sans signes périphériques d'insuffisance cardiaque droite. L'électrocardiogramme basal de repos inscrit une tachycardie sinusale à 120 cpm sans trouble de repolarisation ni hypertrophie pariétale. La radiographie pulmonaire de face visualise une cardiomégalie avec un syndrome interstitiel et des signes de surcharges vasculaires pulmonaires. Sur le plan biologique, on ne note ni trouble ionique (natrémie à 136, kaliémie à 4) ni insuffisance rénale, La troponine et le NT-proBNP sont élevés, respectivement 0,20 ng/ml (N < 0,04 ng/ml) et 500 pg/ml (N < 300 pg/ml). L'échographie cardiaque retrouve un aspect de cardiomyopathie dilatée hypokinétique (Figure 1) avec une altération de la fonction ventriculaire gauche. Le diamètre télédiastolique du ventricule gauche est mesuré à 65 mm, le diamètre télésystolique du ventricule gauche à 51 mm (FE à 35% par Simpson biplan) (Figure 2). Les pressions de remplissages ventriculaires gauches sont élevées avec une insuffisance mitrale de grade II vraisemblablement fonctionnelle. La coronarographie montre un réseau coronaire angiographiquemment sain. Une IRM cardiaque est réalisée : les séquences de ciné-IRM confirment l'hypocinésie globale du ventricule gauche, la FEVG est calculée à 35 %. IL n'y a pas de prise de contraste du myocarde sur les séquences tardives réalisées 10 min après injection de gadolinium L'évolution sous traitement médical associant diuretique de l'anse, Inhibiteur de l'enzyme de conversion et BBloquant est marquée par une amélioration clinique et échocardiographique (FE est évalué à 50% après 6 mois de traitement).

## Discussion

**Figure 3:**
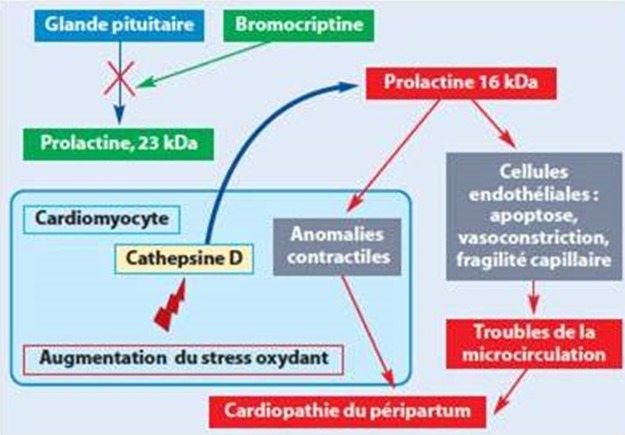
L'hypothèse physiopathologique de la prolactine selon Hilfiker-Kleiner

La cardiomyopathie du péripartum (CMP-PP) est définie par la Société Européenne de Cardiologie (ESC), par « une cardiomyopathie dilatée se manifestant dans la période du péripartum chez une patiente préalablement en bonne santé » [1]. Elle consiste en une dysfonction ventriculaire gauche systolique avec diminution de la fraction d'éjection ventriculaire gauche (FEVG) en échocardiographie, se manifestant dans le dernier mois de grossesse, ou dans les 5 mois suivant l'accouchement [1], son incidence est de 1/1500 à 1/4000 naissances vivantes [2] avec une grande variété géographique. Plusieurs facteurs de risque de MCP-PP ont été identifiés : âge maternel > 30 ans, multiparité, grossesse multiple, obésité, hypertension artérielle, pré-éclampsie, tocolyse prolongée [3]. De nombreuses hypothèses physiopathologiques ont été proposées à savoir une mauvaise adaptation aux modifications hémodynamiques de la grossesse liée à une augmentation du débit cardiaque, à une augmentation du volume plasmatique et à une altération des résistances vasculaires périphériques. Une réponse auto-immune anormale à la grossesse avec l'expression d'autoanticorps cardiaques spécifiques. D'autres études suggèrent l'implication de lésions de myocardite d'origine virale [4]. L'équipe d'Hilfiker-Kleiner a pointé le rôle d'un peptide issu du clivage de la prolactine, hormone de la lactation [5]. Il a, en effet, été démontré, que l'augmentation du stress oxydant intramyocardique augmente l'activité enzymatique de la cathepsine D et son relargage dans la circulation sanguine, responsable du clivage de la prolactine aboutissant à la libération d'un peptide de 16 kDa. Ce peptide a des propriétés anti-angiogénique et proapoptotique, responsable d'une inadéquation de la vascularisation myocardique et d'une dysfonction contractile des cardiomyocytes (Figure 3). Le tableau classique est celui d'une insuffisance cardiaque globale, parfois purement gauche, en générale sévère et d'installation extrêmement rapide, parfois sur quelques heures [4]. Des douleurs thoraciques, présentent dans près de 50 % des cas, soit à type de précordialgies atypiques, soit de type angineuse, voire infarctoïde. L'électrocardiogramme ne montre pas de signes spécifiques, mais parfois un bloc de branche gauche ou des ondes T négatives. L'Échocardiographie transthoracique est l'examen clef, il permet d'affirmer le diagnostic et de surveiller l'évolution de la CMP-PP Elle retrouve une dilatation ventriculaire, une diminution de la fraction d'éjection inférieure à 45 %, une possible atteinte ventriculaire droite associée [6]. Elle recherchera des complications à type de thrombose intracavitaire et d'épanchement péricardique associée Enfin, elle permettra d'éliminer une cardiopathie préexistante (hypertrophique, valvulaire rhumatismale, ischémique). L'IRM cardiaque met en évidence un rehaussement tardif après injection de gadolinium, non systématisé, à prédominance sous-épicardique, et dont l'intensité semble corrélée au pronostic et à la probabilité de récupération fonctionnelle ventriculaire gauche [7].

Le traitement de la CMP-PP est celui de l'insuffisance cardiaque chronique, avec l'association bêtabloquant, Inhibiteur d'enzyme de conversion (IEC) et diurétiques. Dans les formes sévères, il est parfois nécessaire d'avoir recours au traitement inotrope par voie intraveineuse et compte tenu du risque de manifestations thromboemboliques, il est recommandé d'instaurer un traitement par anticoagulant. En cas de récupération ad integrum de la fonction systolique, la poursuite du traitement au long cours est recommandée bien que l'interruption du traitement ne semble pas s'accompagner d'une nouvelle dégradation de fonction systolique [8]. En l'absence de récupération et selon l'évolution clinique, une resynchronisation cardiaque pourra être proposée, une assistance ventriculaire gauche et en derniers recours une transplantation cardiaque. La bromocriptine (anti-prolactine) a fait la preuve de son efficacité reposant sur l'hypothèse physiopathologique du rôle cardiotoxique de la prolactine de petit poids moléculaire comme facteur déclenchant la CMP-PP. Une étude a comparé le traitement par IEC seuls et IEC + bromocriptine, en ouvert, chez 20 femmes. Le diagnostic est porté dans le mois qui suit la grossesse. Les malades reçoivent 2,5 mg de bromocriptine 2 fois par jour pendant 2 semaines puis 1 fois par jour pendant 6 semaines. À six mois, les patientes ayant reçu le traitement par bromocriptine présentaient, par rapport au groupe contrôle : une FEVG mesurée en IRM supérieure, une mortalité inférieure et un taux inférieur de survenue du critère de jugement principal (défini par un décès et/ou une insuffisance cardiaque classe III/IV NHYA et/ou une FEVG < 35 %) [9]. Une contraception efficace est essentielle chez ces femmes jeunes dans les suites d'un premier épisode de CMP-PP. En cas de dysfonction persistante, toute nouvelle grossesse doit être découragée, et contre-indiquée en raison du risque élevé de décès. Si la fonction systolique s'est normalisée, une nouvelle grossesse peut être envisagée mais les risques de complications doivent être clairement exposés. L'évolution clinique des cardiomyopathies du péri-partum est variable. L'importance de la dysfonction ventriculaire gauche initiale (fonction inférieure à 30 %) et de la dilatation ventriculaire (supérieure à 27 mm/m^2^) sont des éléments péjoratifs [10]. Cependant, la prédiction à l'échelle individuelle reste délicate, et il est classique d'observer des récupérations intégrales même chez les patientes ayant une dysfonction systolique initiale sévère [11]. La persistance d'une dysfonction ventriculaire gauche au-delà du sixième mois du post-partum est de mauvais pronostic. Cependant, des données font également état de récupérations intégrales plus tardives, avec des délais de plus de 3 ans [11].

## Conclusion

La cardiomyopathie du péripartum est une complication cardiaque grave de la grossesse. Souvent sous-diagnostiquée et d'origine est multifactorielle, son potentiel évolutif extrêmement rapide et totalement imprévisible justifie une prise en charge multidisciplinaire dans un centre spécialisé afin d'améliorer le pronostic materno-fœtal.
